# Melatonin Levels Decrease in the Umbilical Cord in Case of Intrauterine Growth Restriction 

**DOI:** 10.25122/jml-2020-0128

**Published:** 2020

**Authors:** Andrii Mykolaiovych Berbets, Adrian Mykhailovych Barbe, Oksana Anatoliivna Andriiets, Anatolii Volodymyrovych Andriiets, Oleksandr Mykhailovych Yuzko

**Affiliations:** 1. Department of Obstetrics and Gynecology, Bukovinian State Medical University, Chernivtsi, Ukraine

**Keywords:** Pregnancy, placenta, IUGR, melatonin, cytokines, PlGF, ELISA – enzyme-linked immunosorbent assay, FLT-1 – fms-related tyrosine kinase-1, IL-1-β – interleukine-1-β, IL-4 – interleukine-4, IL-6 – interleukine – 6, IL-10 – interleukine-10, IUGR – intrauterine growth restriction, PI – placental insufficiency, PlGF – placental growth factor, sFLT-1 – soluble fms-like tyrosine kinase-1, TNF-α – tumor necrotizing factor-α, VEGFR – vascular endothelial growth factor receptor

## Abstract

Intrauterine growth restriction (IUGR) is a common reason for perinatal morbidity and mortality. Also, it is often complicated with fetal distress. Melatonin is widely known as an anti-oxidant agent, and it might decrease the damage of tissues caused by hypoxia. It is also known that levels of pro- and anti-inflammatory cytokines are changed during pregnancy. Placental growth factor (PlGF) is responsible for the angiogenesis in the placenta. We aimed to investigate whether the level of melatonin, cytokines, and PlGF in umbilical blood after birth is different in the case of IUGR compared to normal fetuses. Fourteen women whose pregnancies were complicated with IUGR were included in the study group. The presence of IUGR was confirmed by ultrasound fetometry in the third pregnancy trimester, 30-36 weeks of gestation. All patients delivered their children vaginally after 37 weeks of pregnancy. The cases of severe fetal distress that required a caesarian section, obstetrical forceps, or vacuum extraction of the fetus were excluded from the study. We found that the concentrations of cytokines did not differ significantly between the groups. Also, no significant difference in the daytime of delivery was found between the groups. The concentrations of melatonin and PlGF in the umbilical blood at labor were significantly lowered in the case of IUGR compared to normal pregnancies. This fact, as we consider, is caused by altered production of melatonin and PlGF by the placenta. Therefore, the protective action of these two factors for the fetus at labor is decreased in IUGR.

## Introduction

The neuroendocrine system of a pregnant woman plays a crucial role in the successful development of pregnancy. The pineal gland (epiphysis) is one of the most important parts of this system. It produces and secrets such hormones as melatonin and serotonin. Melatonin (5-methoxy-N-acetyltryptamine) chemically belongs to the class of indols, and it easily crosses the blood-brain barrier [1, 2]. The placental melatonin system is present throughout pregnancy and regulates villous trophoblast differentiation [3]; melatonin is involved in successful implantation of a fertilized egg [3], influences the act of delivery [4], decreases oxidative stress in pregnancy [5], especially in case of pre-eclampsia [2, 6, 7]. In our previous studies, we established that melatonin decreases in pregnant women’swomen’s blood with intrauterine fetal growth restriction (IUGR) [8]. The interactions between the pineal gland and a placental trophoblast as between the producers of melatonin are still unclear. But it has been admitted that melatonin can be considered as one of the biochemical markers which depict the conditions of the placenta’splacenta’s tissue.

Placental Growth Factor (PlGF), among others, can also be considered as such a marker. PlGF has a pro-angiogenic effect on the placental tissue; it also stimulates the proliferation of the trophoblast, and it has been known as a predictor and a diagnostic marker of pre-eclampsia [9, 10]. On the other hand, lately, this molecule attracts the attention of researchers in the case of placental insufficiency (PI) or fetal distress. In our recent study [11], we established that concentrations of PlGF in the blood of pregnant women significantly decrease in case of placental insufficiency, manifested as intrauterine fetal growth restriction syndrome. Other studies confirm the fact that the levels of PlGF are lowered in case the hypoxic conditions of a fetus and/or a newborn are eventually diagnosed [12], or if blood flow disorders, expressed as ““ischemic placental syndrome””, are present in the uterine and umbilical arteries [13]. The presence of links between the decrease of PlGF and the expression of PI and IUGR has also been confirmed by other studies [14-16].

The impact of melatonin on the immune system, especially on the cytokine level, is being widely studied nowadays. Melatonin decreases stress-induced inflammation [17] and reduces the inflammatory response in the case of sepsis due to a decrease in the level of interleukin-10 (IL-10) [18]. We also recently found that in the case of IUGR, melatonin concentrations in maternal blood significantly decrease, resulting in strengthening of the pro-inflammatory immunity, shown by the increasing of TNF-α, IL-1-β and IL-6 levels in the blood of pregnant women. The increase in the levels of the anti-inflammatory cytokines, such as IL-4 and IL-10, was also significant in the case of IUGR [8].

Based on the above-mentioned facts, the primary conclusion that can be drawn is that the changed levels of melatonin, PlGF, and cytokines both in the blood plasma of a pregnant woman and umbilical blood might be relevant in the diagnosis of PI and IUGR.

## Material and Methods

### Subjects

The study was conducted on 14 women whose pregnancies were complicated with IUGR. The presence of IUGR was confirmed by ultrasound fetometry in the third pregnancy trimester, at 36 weeks of gestation (the estimated bodyweight of the fetus was below the third percentile for the current pregnancy term). The control group consisted of 13 women who had uncomplicated pregnancies. All patients delivered their children vaginally after 37 weeks of pregnancy. The cases of severe fetal distress, which required a caesarian section, obstetrical forceps, or vacuum extraction of the fetus, were excluded from the study. All the cases of IUGR were confirmed after the birth of fetuses. The umbilical blood was taken immediately after a baby’s birth from the placental side of the clamped and cut umbilical cord before the birth of the placenta.

### Proceeding of biochemical analysis

The concentrations of melatonin and PlGF were assayed using an ELISA kit manufactured by “IB” (Germany); the concentrations of pro-inflammatory (TNF-α, IL-1-β, IL-6) and anti-inflammatory (IL-4, IL-10) cytokines were measured using diagnostic kits manufactured by “Vector Best” (Ukraine).

### Statistical analysis

Statistical data were calculated using the MedCalc software, developed by “MedCalc Software” (Ostend, Belgium). The results were estimated using the Mann-Whitney U-test for small groups. A P-value <0.05 was considered statistically significant.

### Ethics approval

The study was approved by the Biological and Medical Ethics Committee of the Higher State Educational Establishment of Ukraine “Bukovinian State Medical University” (minutes No3, March 30th, 2017), and was carried out following The Code of Ethics of the World Medical Association (Declaration of Helsinki) for experiments involving humans. All the patients involved in the study gave their consent for participation and publication of the study’s results.

We did not find any statistical difference in the daytime of delivery between groups.

## Results

The levels of melatonin in the umbilical blood taken from the patients from the studied groups are represented in Table 1.

**Table 1:** The levels of melatonin in the umbilical blood taken during labor from women with diagnosed IUGR.

	Women with IUGR (n=14)	Women of control group (n=13)	P-value
**Melatonin (pg/ml)**	7.50 (3.0818 – 13.4042)	14.60 (9.58 – 23.79)	0.00101
1. Р-value was calculated accordingly to the Mann-Whitney U-test.			
2. A 95% confidence interval of the median is indicated in brackets.			

**Figure 1:**
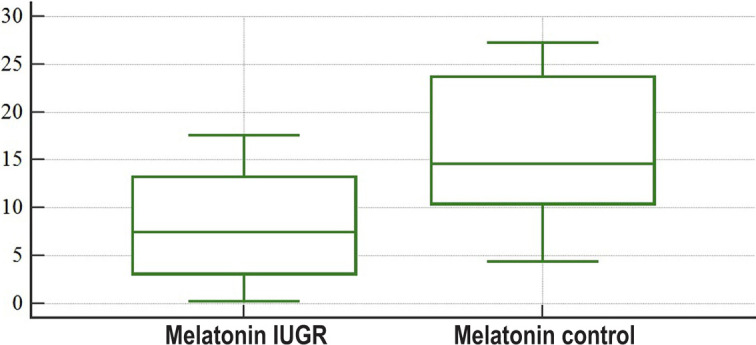
Graphical comparison of melatonin concentrations in the umbilical blood taken after a newborn’s birth from the placental side of the umbilical cord before the placenta’s birth: study group (Melatonin IUGR) and control group (Melatonin control).

As can be seen in Table 1, the level of melatonin in the umbilical blood, taken after a newborn’s birth from the placental side of the umbilical cord before the birth of the placenta, was significantly lower (Р=0.0101) compared to healthy women (Figure 1). Therefore, we can affirm that in the case of IUGR, the levels of melatonin in fetal blood are decreased at birth.

The levels of PlGF in the umbilical blood taken from the patients in the studied groups are represented in Table 2.

**Table 2:** The levels of PlGF in the umbilical blood taken during labor from women with diagnosed IUGR.

	Women with IUGR (n=14)	Women of control group (n=13)	P-value
**PIGF (pg/ml)**	99.15* (84.38 – 153.92)	155.30 (98.78 – 354.21)	0.0427
1. Р-value was calculated accordingly to the Mann-Whitney U-test.			
2. A 95% confidence interval of the median is indicated in brackets.			

**Figure 2:**
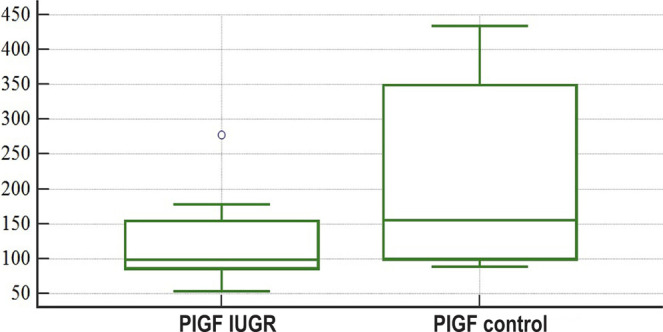
Graphical comparison of PlGF concentrations in the umbilical blood, taken after a newborn’s birth from the placental side of the umbilical cord before the birth of the placenta: study group (PlGF IUGR) and control group (PlGF control).

It has been established that the concentration of PlGF in the umbilical blood taken during labor from women whose pregnancies were complicated with IUGR was 1.57 times lower than women who had uncomplicated pregnancies, p<0.05 (Figure 2).

The levels of pro-inflammatory cytokines taken from the umbilical blood during labor from the examined patients are represented in Table 3.

**Table 3:** The levels of TNF-α, IL-1-β, IL-6 in the umbilical blood taken during labor from women with diagnosed IUGR.

	Women with IUGR (n=14)	Women of control group (n=13)	P-value
**TNF-α** **(pg/ml)**	28.55 (9.77 – 13.40)	40.30 (28.49 –68.80)	0.1694
**IL-1-β** (pg/ml)	25.75 (18.94 – 37.32)	38.00 (22.80 – 56.24)	0.1821
**IL-6 (pg/ml)**	38.60 (30.17 – 50.04)	37.20 (30.97 – 61.40)	0.7564
1. Р-value was calculated accordingly to the Mann-Whitney U-test.			
2. A 95% confidence interval of the median is indicated in brackets.			

Data represented in Table 3 shows that there was no clear difference in the concentrations of pro-inflammatory cytokines between the studied groups. In the IUGR group, we found just a tendency of decrease of TNF-α and IL-1-β compared to patients with uncomplicated pregnancies, and IL-6 levels were practically equal in both groups.

The results of studying the levels of anti-inflammatory cytokines in the umbilical blood sampled during labor from women in the studied groups are represented in Table 4.

**Table 4:** The levels of IL-4 and IL-10 in the umbilical blood taken during labor from women with diagnosed IUGR.

	Women with IUGR (n=14)	Women of control group (n=13)	P-value
**IL-4 (pg/ml)**	7.45 (4.38 – 14.19)	6.30 (3.30 – 8.65)	0.3440
**IL-10 (pg/ml)**	2.10 (0.98 – 6.26)	1.70 (0.00 – 2.95)	0.3181
1. Р-value was calculated accordingly to the Mann-Whitney U-test.			
2. A 95% confidence interval of the median is indicated in brackets.			

Based on the data represented in Table 4, we can assert that no significant difference was found in the concentrations of anti-inflammatory cytokines between the studied groups.

**Figure 3:**
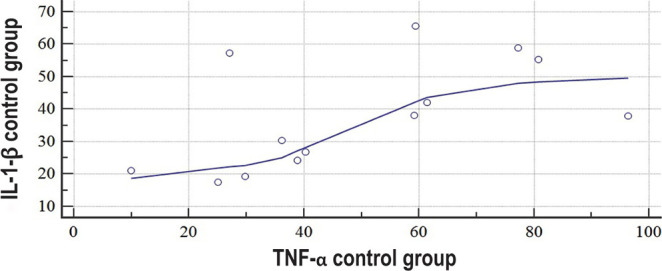
Scatter diagram of data distribution and correlation graph between the concentrations of IL-1-β and TNF-α in the umbilical blood during labor in women who had uncomplicated pregnancies.

Analysis of correlation links between studied indexes showed a moderate correlation between IL-1-β and TNF-α in the group of women who had uncomplicated pregnancies (r= 0.5738, P=0.0403, Figure 3).

**Figure 4:**
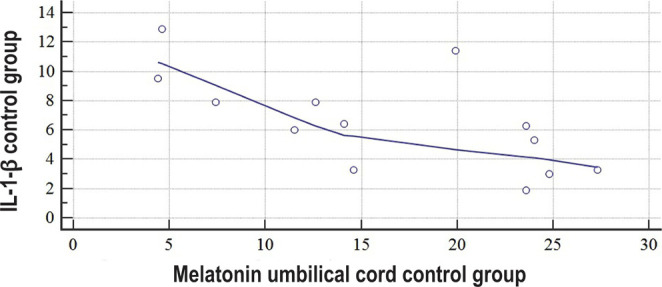
Scatter diagram of data distribution and correlation graph between the concentrations of IL-1-β and melatonin in the umbilical blood during labor in women who had uncomplicated pregnancies.

We also established a moderate negative correlation link between the concentrations of anti-inflammatory cytokine IL-4 and melatonin in the group of patients with uncomplicated pregnancies (r= -0.6587, P=0.0143, Figure 4).

**Figure 5:**
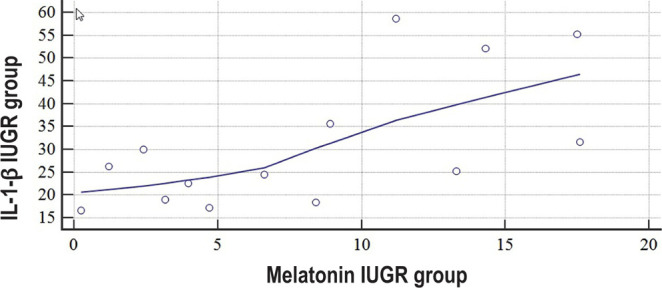
Scatter diagram of data distribution and correlation graph between the concentrations of IL-1-β and melatonin in the umbilical blood during labor in women who had pregnancies complicated with IUGR.

In the group of patients whose pregnancies were complicated with IUGR, we established a moderate correlation link between melatonin and pro-inflammatory cytokine IL-1-β levels (r= 0.6565, P=0.0108, Figure 5).

## Discussion

In our study, we found that the level of melatonin in the umbilical blood taken during the third period of labor from pregnant women whose pregnancies were complicated with IUGR is considerably decreased compared to the patients with uncomplicated pregnancies. Therefore, the level of melatonin in the fetal blood at labor is significantly lowered in the case of IUGR. It has been established that the placenta actively produces melatonin [3]. Production of melatonin significantly decreases in case of placental insufficiency, which manifests as IUGR [8], and the pineal glands of both mother and fetus seem to be unable to compensate for the deficiency of melatonin, which appears in case of PI.

Secondly, PlGF is also decreased in umbilical blood in case if the pregnancy is complicated with IUGR. There are papers that mention changes in the concentrations of PlGF in maternal blood, mostly in the case of pre-eclampsia [9-12]. Very few papers pay attention to the changes in the levels of PlGF in the case of IUGR, especially in the umbilical blood [14]. PlGF is responsible for angiogenesis in the placenta and actively binds to the vascular endothelial growth factor-1 receptor-1 (VEGFR-1), also known as fms-related tyrosine kinase-1 (FLT-1) and its soluble variant, soluble fms-like tyrosine kinase-1 (sFLT-1). Stimulation of FLT-1 causes angiogenesis and collateral growth in ischemic tissues [15]. Therefore, this process is violated in the case of PI, which manifests as IUGR.

Despite not finding the statistically significant difference between the umbilical cord concentrations of the studied cytokines in the groups (probably due to the small number of patients involved), we established that melatonin has a moderate correlation with one of the main pro-inflammatory cytokines, namely IL-1-β, if the pregnancy is complicated with IUGR. In the control group, this correlation was also moderate but negative. Based on this fact, we suggest that melatonin plays the role of a moderator in the inflammatory reaction of the placental tissue, which is clearly expressed in the case of PI, manifesting as IUGR [8]. Anti-inflammatory and anti-oxidative actions of melatonin are widely known [5, 6], and they cannot be well enough expressed in the case of IUGR.

Our study has certain strengths, such as the strict inclusion criteria (the diagnosis of IUGR was confirmed in all cases included in the study group), sampling of the umbilical cord was performed during the third period of labor, immediately after the birth of the fetus (this non-invasive procedure allowed us to judge about the concentrations of the studied biochemical markers in the fetal blood at birth) and estimation of correlation links between studied indexes. However, it was also limited by the small number of patients included in the study.

## Conclusion

The concentrations of melatonin and PlGF in the umbilical blood during labor are significantly lower in the case of IUGR compared to normal pregnancies. This fact, as we consider, is caused by the altered production of melatonin and PlGF by the placenta. Therefore, the protective action of these two factors for the fetus during labor is decreased in the case of IUGR.

## Conflict of Interest

The authors declare that there is no conflict of interest.
